# Integrative Analysis of Multi-Omics Data-Identified Key Genes With KLRC3 as the Core in a Gene Regulatory Network Related to Immune Phenotypes in Lung Adenocarcinoma

**DOI:** 10.3389/fgene.2022.810193

**Published:** 2022-03-31

**Authors:** Kai Mao, Yunxi Zhao, Bo Ding, Peng Feng, Zhenqing Li, You Lang Zhou, Qun Xue

**Affiliations:** ^1^ Cardiovascular Surgery Department, Affiliated Hospital of Nantong University, Nantong, China; ^2^ Research Center of Clinical Medicine, Affiliated Hospital of Nantong University, Nantong, China; ^3^ Department of Neurology, Rugao People’s Hospital, Nantong, China; ^4^ Neurosurgery, Shaanxi Provincial People’s Hospital, Shaanxi, China

**Keywords:** lung adenocarcinoma, PD-1 inhibitor, LASSO analysis and SVM-RFE, immune cell infiltration, TCGA

## Abstract

In a recent study, the PD-1 inhibitor has been widely used in clinical trials and shown to improve various cancers. However, PD-1/PD-L1 inhibitors showed a low response rate and were effective for only a small number of cancer patients. Thus, it is important to figure out the issue about the low response rate of immunotherapy. Here, we performed ssGSEA and unsupervised clustering analysis to identify three clusters (clusters A, B, and C) according to different immune cell infiltration status, prognosis, and biological action. Of them, cluster C showed a better survival rate, higher immune cell infiltration, and immunotherapy effect, with enrichment of a variety of immune active pathways including T and B cell signal receptors. In addition, it showed more significant features associated with immune subtypes C2 and C3. Furthermore, we used WGCNA analysis to confirm the cluster C-associated genes. The immune-activated module highly correlated with 111 genes in cluster C. To pick candidate genes in SD/PD and CR/PR patients, we used the least absolute shrinkage (LASSO) and SVM-RFE algorithms to identify the targets with better prognosis, activated immune-related pathways, and better immunotherapy. Finally, our analysis suggested that there were six genes with KLRC3 as the core which can efficiently improve immunotherapy responses with greater efficacy and better prognosis, and our study provided clues for further investigation about target genes associated with the higher response rate of immunotherapy.

## Introduction

According to recent research, lung cancer is a highly malignant type of cancer with poor prognosis. It ranks among the top cancers in terms of morbidity and mortality (Jemal et al., 2011), wherein about 60% of the patients in the early stages of lung cancer underwent combined treatment of surgery, chemotherapy, and radiotherapy but did not achieve satisfactory therapeutic effect, and cancer-driver gene-targeted therapy also encounters the problem of drug resistance [27]. The emergence of immunotherapy brings hope to lung cancer patients. Also, the interactions of PD-1 and its ligands constitute a major immunosuppressive pathway in tumors (Brahmer et al., 2012; Robert et al., 2015). Since 2010, the PD-1 or PD-L1 antibodies have been showing significant antitumor activity, including NSCLC (Ohe et al., 2007; Herbst et al., 2015). In addition to activating T-cells by binding to its ligands (PD-L1 and PD-L2) to enhance T-cell antitumor functions, the PD-1 effect was also seen in other immune cells (Barber et al., 2006; Sharpe et al., 2007).

Nowadays, several therapeutic antibodies with PD-1- or PD-L1-suppressing properties have been formulated for managing malignancies such as NSCLC clinically. However, these immunotherapies show a low remission rate in patients. Thus, a great deal of effort has been undertaken to find predictive biomarkers for patients with optimal response to these inhibitions, and there were also studies devoted to finding biological targets to improve the response rate of immunotherapy. Hu et al. analyzed the differences in the gene expression profiles of patients with high and low expression of PD-1 and PD-L1 and found that more than one hundred genes, including IL-21, KLRC3, and KLRC4, were significantly upregulated in the high-expression group, compared with the low-expression group [26]. In the present, it is urgent to confirm the targets that can be applied to improving the response rate of immunotherapy.

Here, we performed the ssGSEA and unsupervised clustering analysis to identify targets with better prognosis, immunotherapy, and that are activated in immune-related pathways based on the red module for 111 genes that are highly correlated with cluster C based on the LASSO and SVM-RFE analyses. The six genes with KLRC3 as the core were identified as the key genes with a better prognosis and correlated with immunotherapy.

## Materials and Methods

### Datasets and Samples

The gene expressions of a total of 1881 patients with detailed survival information obtained from TCGA-LUAD (https://portal.gdc.cancer.gov/) and GEO datasets of GSE31210, GSE30219, GSE68465, GSE37745, GSE50081, and GSE72094 were generated. The expression values were log-transformed, and the “ComBat” algorithm was used for reducing probable batch effects resulting from the inter-dataset biases (non-biotech) (Johnson et al., 2007).

### Gene Signature and Single-Sample Gene Set Enrichment Analysis

A set of marker genes for types of immune cells was selected based on Bindea et al. (2013). For enrichment computation in the individual sample gene set, the absolute enrichment fractions were derived via the GSEA program for traits that have been validated by prior experimentation. For confirming the immune cell populations, the ssGSEA analysis of each sample was accomplished using the immune cell signature gene predictions (Supplementary Table S1) (Bindea et al., 2013).

### Gene Set Variation Analysis and Functional Annotation

To explore the biological event differences between clusters A, B, and C, we used the “GSVA” R packages to conduct the GSVA enrichment analysis (Figure 2A). The “c2.cp.kegg.v7.2.symbols.gmt” was obtained from the MSig DB dataset, and the *p*-adjust < 0.5 was considered as statistically significant. The subtypes also were seen to correlate with the immune studies and prognosis by Thorsson et al. (2018), aggressive subtype by Dama et al. (2017), and luminal and basal subtypes by Zhao et al. (2019) to analyze the overlap with our study cluster C (Figure 3A). To confirm the events in the different subgroups, the distribution of mutant gene frequencies affected by SNVs and CNVs was investigated across various subtypes (Figure 3B). The frequency of mutations in each gene was significantly different across subtypes (Fisher exact test with the BH test correction, adjusted *p* < 0.05).

### Consensus Clustering for Tumor-Infiltrating Immune Cells and Differential Expression Genes

For each sample, hierarchical agglomerative clustering was implemented for LUAD depending on a specific pattern. In this procedure, the “Consensus Cluster Plus” R package was used to perform “PAM” analysis, which is a Euclidean distance and Ward’s linkage-based unsupervised clustering approach. To ensure clustering stability, the aforementioned process was repeated about 100 times.

### Differential Expression Genes Associated With the Two Clusters

Depending on the infiltration of immune cells, patients were classified into high and low immune-cell infiltration subtypes. To determine DEGs between two clusters, the limma R package was utilized, and absolute fold change was designated to >1, and significance criterion adjusted to *p* < 0.05.

### Construction of Signature Gene of Lung Adenocarcinoma

For immunotherapy response assessment of lung cancer patients presenting newly defined immunophenotypes, the gene expression profiles and clinical outcome data of 348 patients from the IMvigor210 (a clinical response trial dealing with PD-L1 blockade by atezolizumab) were collected. The responses to anti-PD-L1 therapy constituted the observed endpoints, which were complete response (CR), partial response (PR), progression of disease (PD), and stability of disease (SD). Regarding the objective response rate (ORR) and disease control rate (DCR), they, respectively, involved patients with CR and PR (for ORR) and patients with CR, PR, and SD (for DCR). Based on the top 10 marked genes of metabolic subtypes, we separated the IMvigor210 cohort into three subtypes (cluster A, cluster B, and cluster C).

For candidate gene selection, we used a least absolute shrinkage and selector operation (LASSO) algorithm, whose penalty parameter was adjusted by setting a cross-validation (10-folds) approach. Meanwhile, we used another algorithm, support vector machine–recursive feature elimination (SVM-RFE), to accomplish gene selection for the CR/PR and SD/PD patients. For further narrowing on the gene among the training cohort, L1-penalized Cox analysis was eventually carried out through gene integrations from either of the aforementioned two algorithms.

### Gene Expression Data With Immunotherapy

Under the Creative Commons 3.0 license (http://research-pub.gene.com/IMvigor210CoreBiologies), the IMvigor210 dataset was obtained from accessible, well-documented software and data package. To determine the status of binary response in various clusters, 298 urothelial cancer patients and 80 immunotherapy recipients with cutaneous melanoma having complete clinical records were analyzed.

### Statistical Analysis

GraphPad and R 4.0.0. were used for all statistical analyses. The Wilcoxon test was conducted for pairwise comparison analysis, and the Kruskal–Wallis test was adopted for comparison among more than two groups. The FDRs in limma and GSEA were adjusted by the Benjamini–Hochberg approach with a significance level of *p* < 0.05. The correlation of categorical clinical information with defined clusters was statistically examined by Fisher’s exact test. All statistical differences were considered significant when *p*-value < 0.05.

## Results

### Identification of Different Subtypes

**FIGURE 1:**
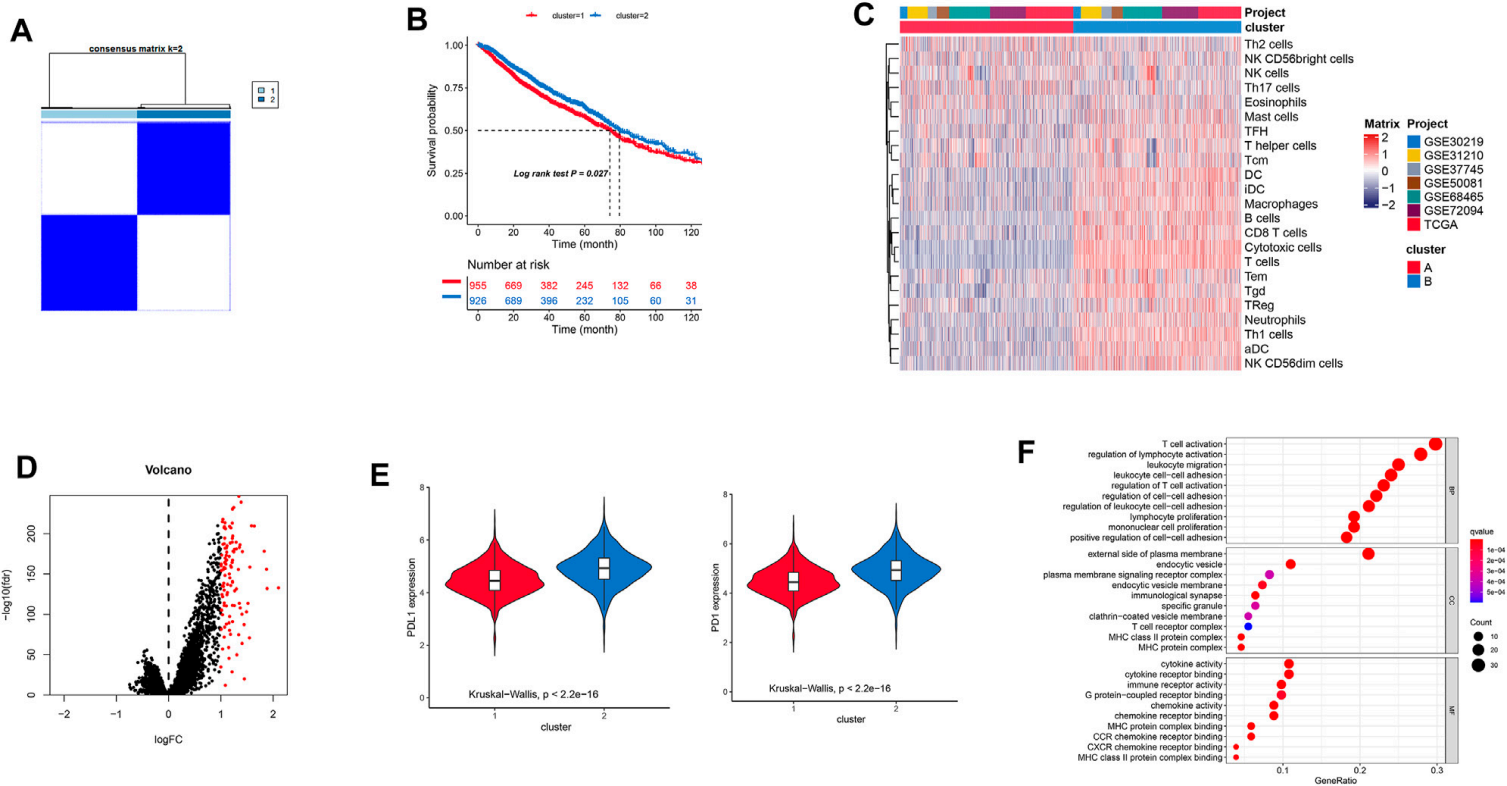
**(A)** Consensus clustering-based identification of two clusters (*n* = 1,881). Sample consensus is displayed by heatmaps shown in white (consensus value = 0) for samples that never aggregated jointly and blue color (consensus value = 1) for samples that always aggregated jointly. **(B)** Survival analysis of patients with two clusters. **(C)** Landscape immune cell infiltration in two clusters. **(D)** Differential gene expressions are imaged by a volcano plot. **(E)** Different expressions of PD-1/PD-L1 in the two clusters. **(F)** GO analysis showing differential gene expressions.

In this study, the immune cell infiltration matrix was used to identify two clusters with different survival rates (Figures 1A,B), and cluster one showed high immune cell infiltration (Figure 1C) such as the DC, B-cells, CD8 T-cells, cytotoxic cells, DC, iDC, macrophages, mast cells, neutrophils, NK, CD-56 dim cells, T-cells, T-helper cells, Tcm, Tem, TFH, Tgd, Th1-cells, and T-Reg. Furthermore, the PD-1 and PD-L1 showed high expression in cluster 2 (Figure 1E). The differential expressions of 110 genes between the two clusters (Figure 1D) were enriched in various immune-related pathways such as T-cell activation and cytokine activity (Figure 1F).

### Identification of Gene Subtypes and Association With Known Subtypes

With the aid of the limma package, the differential expression genes (DEGs) analyses for transcriptome evolution investigation among these clusters performed to identify the biological function of different clusters showed 110 differential gene expressions. For the elimination of redundant genes, the Cox analysis was performed to collect significantly correlated prognosis genes.

**FIGURE 2:**
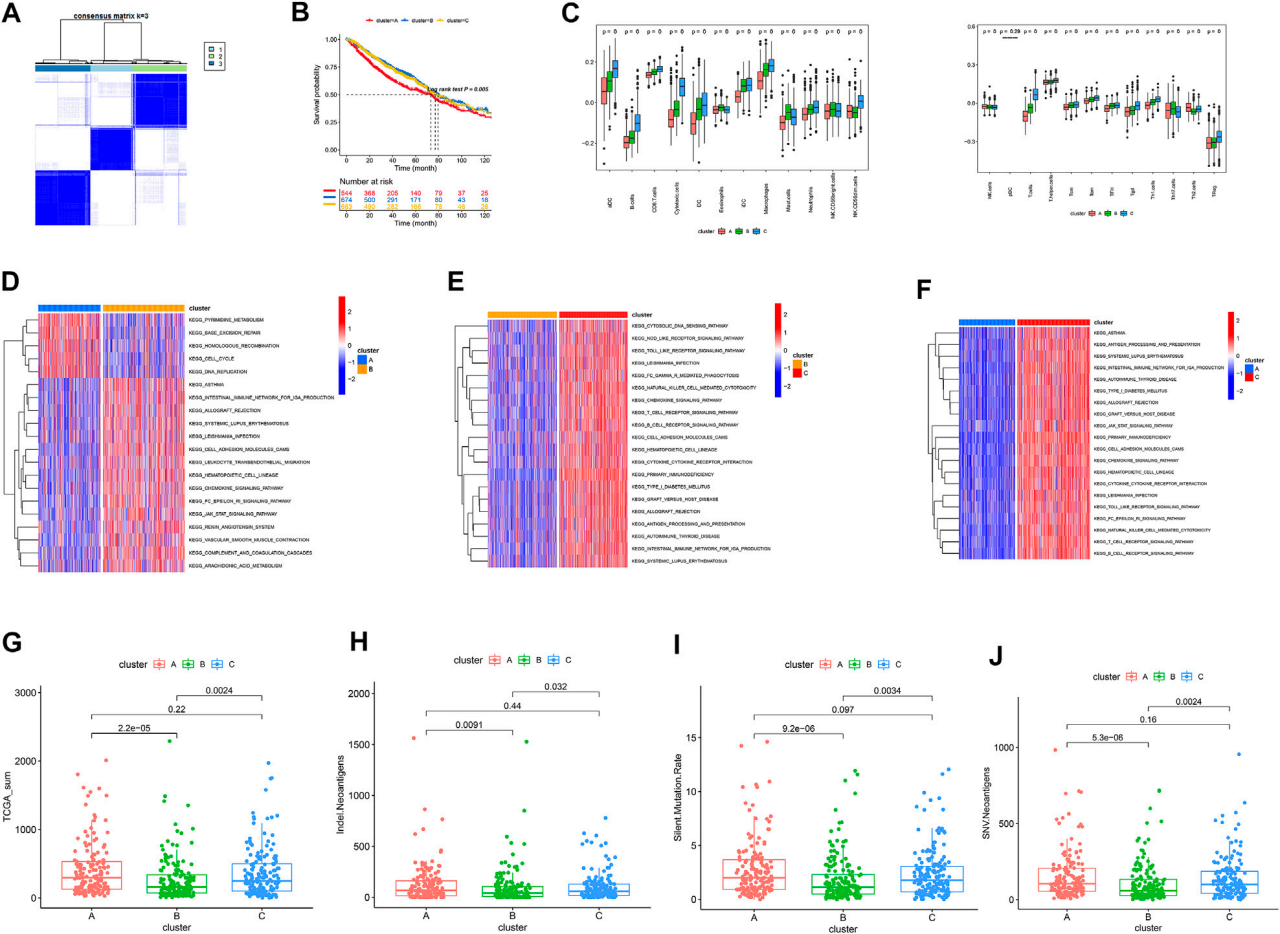
**(A)** Identification of three clusters by consensus clustering. **(B)** Patient survival analysis. **(C)** Landscapes of immune cell infiltration. **(D–F)** KEGG analysis. **(G–J)** Distribution of TMB, neo-antigens (Indel, SNV), and silent mutation rate in the three clusters.

The survival records used to assess the prognostic implication of the clusters (Figure 2B) showed clusters B and C to have a better survival rate than cluster A (*p* = 0.005). Meanwhile, cluster C showed greater immune cell infiltrations such as aDC, B cells, CD8 T cells, cytotoxic cells, DC, eosinophils, iDC, macrophages, mast cells, neutrophils, NK CD56-dim cells, T cells, Tcm, Tem, TFH, Tgd, and Th1 cells (Figure 2C). Furthermore, enrichment of cluster C showed multiple immune-associated pathways, including those for signaling T- and B-cell receptors (Figures 2D–F). Additionally, cluster C showed high tumor mutational burden, neo-antigen (indel and SNV), and PD-1 expression than other subtypes (Figures 2H–K).

**FIGURE 3:**
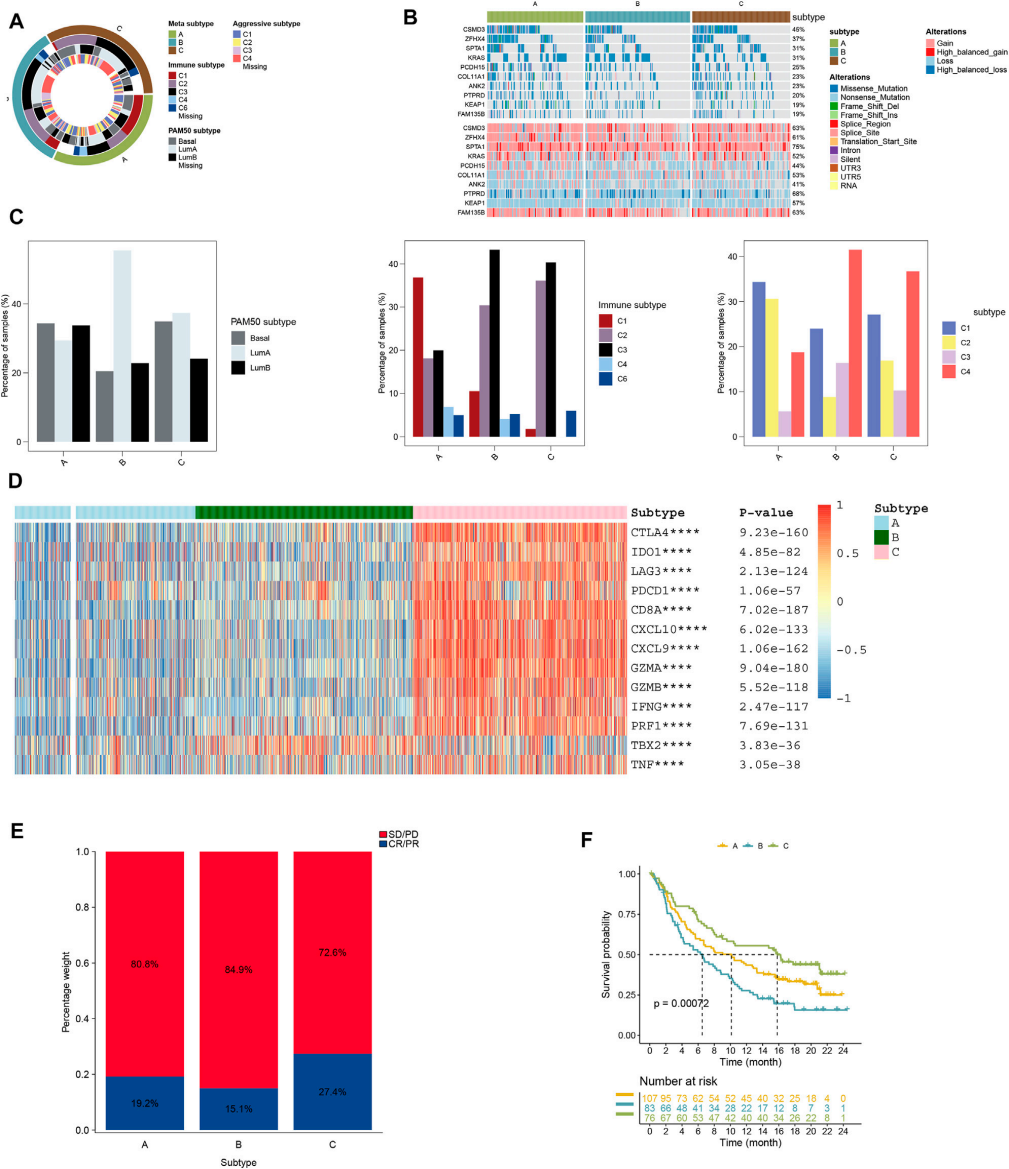
**(A)** Overlay of different clusters (inner ring) with LUAD expression subtypes (outer ring). **(B)** Oncoprint distributions of somatic mutation (SNV/indel) and copy number variation (CNV) events in different clusters. **(C)** Distribution of immune, aggressive luminal, and basal subtypes in different clusters. **(D)** Distribution of immune-related gene expression in different clusters. **(E)** Rate of clinical response to anti-PD-L1 immunotherapy in different clusters, and **(F)** Kaplan–Meier curves for samples with different clusters in the IMvigor210 cohort.

Our findings showed clusters B and C to have high-immune subtypes C3 and C2, high frequency of LumB subtype, and aggressive subtype C4 (Figure 3C). Of these subtypes, the immune subtype C3, LumA subtype, and aggressive subtype C4 found in our study (Figure 2B) were consistent with the findings of others for a better prognosis. In addition to this, a majority of immune-checkpoint-relevant signatures (CD274, CTLA4, HAVCR2, IDO1, LAG3, and PDCD1) and immune-activity-relevant signatures (CD8A, CXCL9, CXCL10, GZMA, GZMB, IFNG, PRF1, TBX2, and TNF) exhibited significant high expression in cluster C (Figure 3D).

### Immunotherapy Response

Our findings also showed that the cluster A subtype had high SD/PD with 72.6% and low CR/PR with 27.4%; however, the cluster A subtype had higher CR/PR with 19.2% than the cluster C subtype (Figure 3F). In the IMvigor210 cohort, the cluster C subtype had a higher survival rate than other subtypes from anti-PD-1 treatment (Figure 3E).

### WGCNA Analysis

**FIGURE 4:**
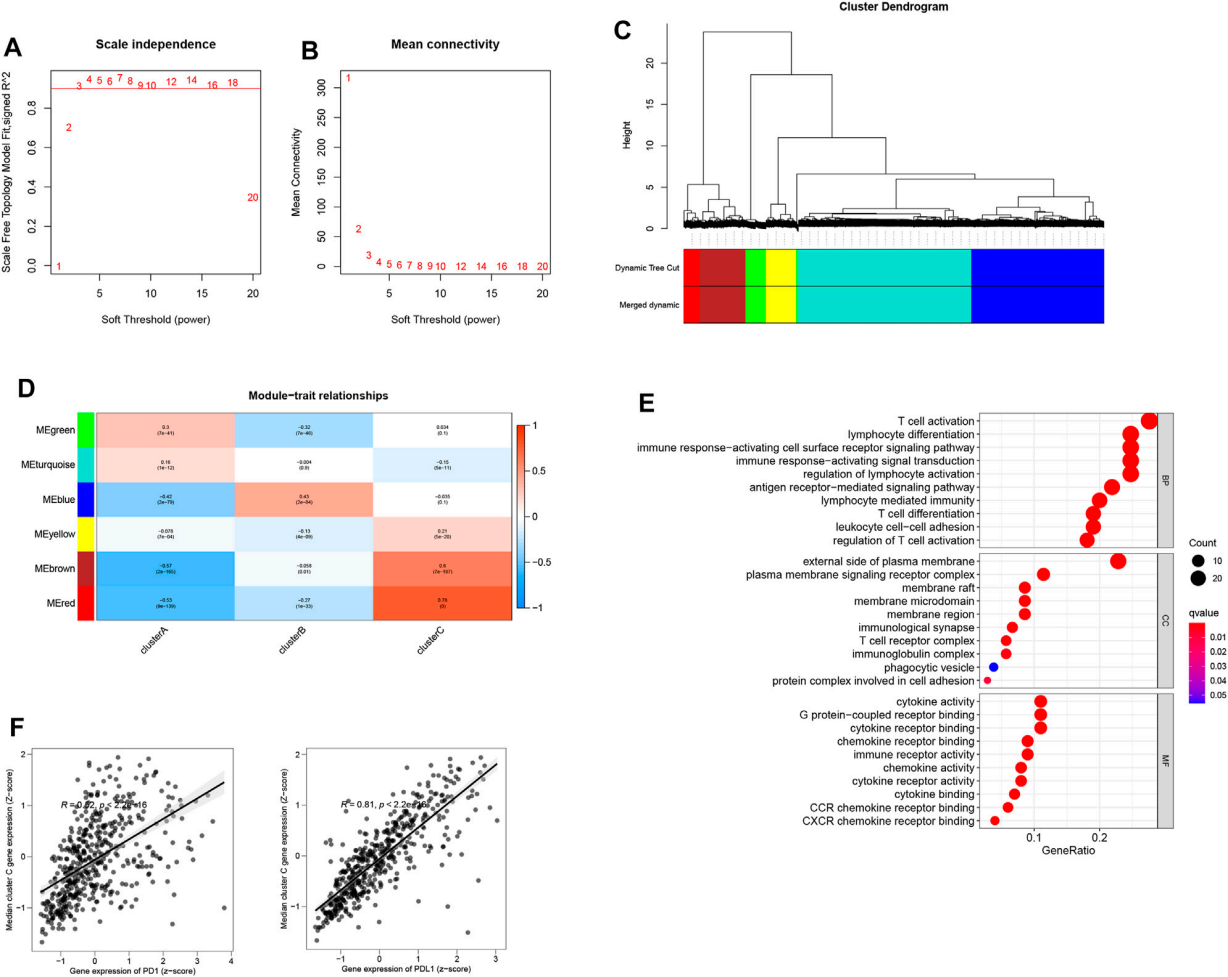
**(A)** Scale-free fit index analysis of 1–20 soft threshold power β. **(B)** Mean connectivity analysis of 1–20 soft threshold power. **(C)** Genes are hierarchically clustered into various modules indicated by different colors. **(D)** Heatmap displaying correlations among module eigengenes. **(E)** GO analysis of the red module genes. **(F)** Correlation of the median expression of genes (red module) with the PD-1/PD-L1 level.

Aided by the R package “WGCNA”, the co-expression network was created from the expression levels of 11,518 genes (Langfelder and Horvath, 2008). Clustering of 1,881 samples was performed by calculating the mean linkage and Pearson’s correlation coefficient. The soft threshold power was set at β = 3 and scale-free R2 = 0.96 to ensure that the scale-free network was constructed (Figures 4A,B). A dynamic mixing and cutting technique was employed to establish the hierarchical clustering tree, each of whose leaves was used to refer to a gene. Meanwhile, a tree branch constituted gene assemblies resembling expression data that were used to refer to a gene module. In this study, a total of six modules were produced (Figures 4C,D), of which the red module showed a high correlation with cluster C from 111 genes enriched by T-cell activation, cytokine activity, and regulation of T-cell activation (Figure 4E). The median expressions of red module genes showed a high correlation with PD-1/PD-L1 expression (Figure 4F).

### Identification of Predictive Signature

**FIGURE 5:**
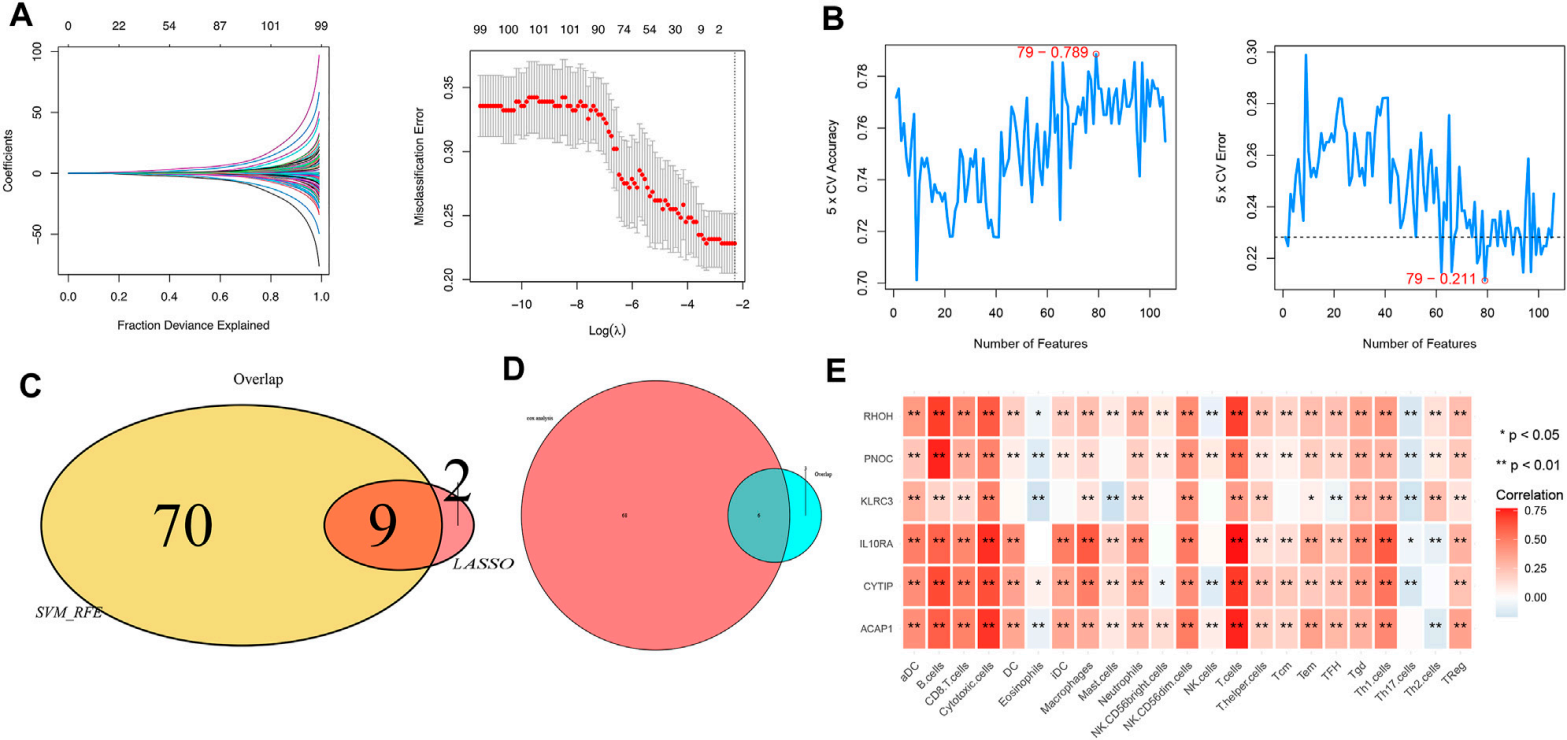
**(A)** LASSO and **(B)** SVM-RFE algorithms in the detection cohort. **(C)** Overlap of incorporated genes selected from two algorithms in the detection cohort. **(D)** Intersection of characteristic genes with PD/RD and Cox analysis genes. **(E)** Correlation between immune cell infiltration and selected genes.

**FIGURE 6:**
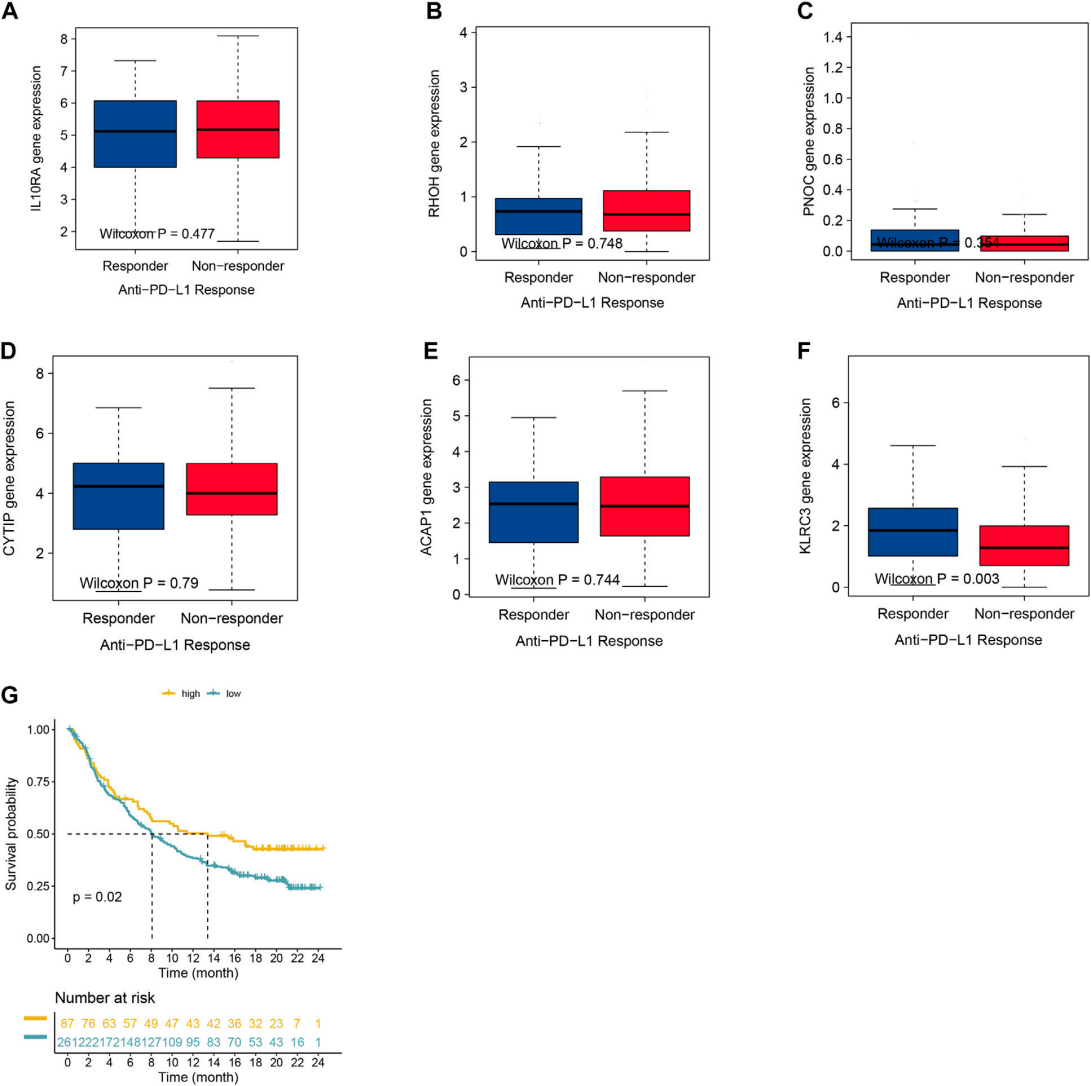
**(A–F)** Expression of selected genes with varying anti-PD-1 responses. **(G)** Kaplan–Meier graphs of KLRC3 expression in the IMvigor210 cohort.

Furthermore, the most significant genes were selected via two algorithms from the CR/PR and SD/PD patients, and the LASSO algorithms were used to identify the prognosis gene. A total of 106 gene candidates were identified after the integration of LASSO- and SVM-RFE-selected genes, of which six genes were selected by both algorithms (Figures 5A–C). The correlations were seen between the overlapping genes, including the PNOC, RHOH, ACAP1, CYTIP, IL10RA, and KLRC3 along with the immune cell infiltrations (Figure 5D). The expression of KLRC3 showed significant response/nonresponse of PD-L1 (Figures 6A–F.) and better survival (Figure 6G).

## Discussion

With the development of immunotherapy, PD-1/CTLA4 inhibitors have been widely used in clinical applications and improved prognosis of various cancers. However, the effective response rate of PD-1 inhibitors in cancer patients, including lung cancer, is low, which is thought to be mainly affected by tumor mutational burden (Goodman et al. (2017), microsatellite instability (MSI), efficient DNA mismatch repair (DMMR) (Xiao and Freeman, 2015), and other factors. In order to improve the response rate of immunotherapy, researchers are constantly working on the study of marker genes and potential biological therapeutic targets related to immunotherapy response. It has been reported that IFN-γ-related gene expression contributes to immunotherapy prediction, such as CCR5, CXCL9, IFNG, STAT1, and PRF1 (Ayers et al., 2017), and these genes are related to tumor antigen presentation, T cytotoxic activity, and immune cell infiltration. These IFN-γ-related genetic signatures are necessary for immunotherapy response (Sharma and Allison, 2015). However, the immune response marker genes for lung adenocarcinoma need to be further studied.

Here, by analyzing the gene expression data of a large number of lung adenocarcinoma patients, we searched for genes associated with better prognosis and immune cell infiltration, hoping to be further used in immunotherapy. Based on differential gene expression, we identified three subtypes showing different survival and immunotherapy effects. Among them, immune-related genes include CD274, CTLA4, HAVCR2, IDO1, LAG3, and PDCD1 as immune checkpoint-related markers, and CD8A, CXCL10, CXCL9, GZMA, GZMB, IFNG, PRF1, TBX2, and TNF (immune activity-related signatures) expressions in cluster C were higher than other subtypes, indicating that cluster C has a high level of immunoreactivity, that is, subtype C has a high potential for immune activation, which can elicit an effective immune response.

Ultimately, six genes including PNOC, RHOH, ACAP1, CYTIP, IL10RA, and KLRC3 were selected by both algorithms of LASSO and SVM-RFE analysis. Prepronociceptin (PNOC) is a preprotein of a series of intracellular products involved in pain and inflammation signaling (Rodriguez-Romaguera et al., 2020). In cancer research, PNOC is highly expressed in glioma cells, epithelial ovarian cancer, and other tumors and has been reported as a prognostic biomarker (Chan et al., 2012; Jin et al., 2018). RHOH, a member of the RhoE/Rnd3 subfamily of GTPases, is highly expressed in B cells, suggesting that it is involved in the development of B-cell malignant leukemia (Dallery-Prudhomme et al., 1997) and is closely related to the immune microenvironment in chronic leukemia (Troeger et al., 2012). ACAP1 is involved in cell membrane transport and cell migration, is an Arf6 GAP, that is, important for immune cell migration and infiltration, and is associated with tumor immune infiltration and prognosis in breast cancer (Zhang et al., 2020). Cytohesin-interacting protein (Cytip) is associated with dendritic cell (DC) maturation and T cell activation and may function in the tumor immune microenvironment (Heib et al., 2012). IL10RA encodes a receptor molecule for the inflammatory factor IL10 and is associated with IL10 expression and STAT3 phosphorylation in colorectal cancer. The expression of IL10RA is also considered to be associated with the clinical stage of colorectal cancer (Zadka et al., 2018).

Most importantly, patients with high expression of KLRC3 had significantly higher response rates to immunotherapy and better prognosis, and KLRC3 can be regarded as the core gene of these six genes. KLRC3 is a natural killer cell receptor gene, and previous studies have shown that KLRC3 affects the stemness and proliferative potential of glioma cells and is involved in glioblastoma tumorigenesis and progression (Cheray et al., 2017). This gene is also upregulated in patients with high expression of PD1 and PDL1 (Hu et al., 2020; Lamberti et al., 2020), suggesting that it may be involved in the regulation of PD1 and PD-L1 expression.

We used WGCNA analysis to confirm cluster C-associated red modules with 111 genes. LASSO and SVM-RFE algorithms confirmed that the key gene KLRC3 was significantly associated with CR/PR status with better prognosis. Our study also has some limitations, such as the lack of experimental and clinical validation data, although the omics data analysis was performed; so, our study results will stimulate further verification or falsification in the scientific community.

Based on our findings in this study, specific changes in the landscape of immune cell infiltration and mutations and transcriptome profiles may have a dramatic impact on improving immunotherapy. KLRC3 may be a core gene suitable for prognosis in lung adenocarcinoma and is associated with SD/PD and CR/PR patients, which will improve reference for future immunotherapy-related studies.

## Data Availability

The original contributions presented in the study are included in the article/Supplementary Material, further inquiries can be directed to the corresponding author.
